# Impact of Pediatric Chronic Dialysis on Long-Term Patient Outcome: Single Center Study

**DOI:** 10.1155/2016/2132387

**Published:** 2016-08-15

**Authors:** Daniella Levy Erez, Irit Krause, Amit Dagan, Roxana Cleper, Yafa Falush, Miriam Davidovits

**Affiliations:** ^1^Institute of Nephrology, Schneider Children's Medical Center of Israel, 49202 Petah Tikva, Israel; ^2^Sackler Faculty of Medicine, Tel Aviv University, 6997801 Tel Aviv, Israel

## Abstract

*Objective.* Owing to a shortage of kidney donors in Israel, children with end-stage renal disease (ESRD) may stay on maintenance dialysis for a considerable time, placing them at a significant risk. The aim of this study was to understand the causes of mortality.* Study Design*. Clinical data were collected retrospectively from the files of children on chronic dialysis (>3 months) during the years 1995–2013 at a single pediatric medical center.* Results.* 110 patients were enrolled in the study. Mean age was 10.7 ± 5.27 yrs. (range: 1 month–24 yrs). Forty-five children (42%) had dysplastic kidneys and 19 (17.5%) had focal segmental glomerulosclerosis. Twenty-five (22.7%) received peritoneal dialysis, 59 (53.6%) hemodialysis, and 6 (23.6%) both modalities sequentially. Median dialysis duration was 1.46 years (range: 0.25–17.54 years). Mean follow-up was 13.5 ± 5.84 yrs. Seventy-nine patients (71.8%) underwent successful transplantation, 10 (11.2%) had graft failure, and 8 (7.3%) continued dialysis without transplantation. Twelve patients (10.9%) died: 8 of dialysis-associated complications and 4 of their primary illness. The 5-year survival rate was 84%: 90% for patients older than 5 years and 61% for younger patients.* Conclusions.* Chronic dialysis is a suitable temporary option for children awaiting renal transplantation. Although overall long-term survival rate is high, very young children are at high risk for life-threatening dialysis-associated complications.

## 1. Introduction 

End-stage renal disease (ESRD) is a major cause of morbidity and mortality in children. In the pediatric population, ESRD is mainly due to congenital anomalies of the kidney and urinary tract (CAKUT) and glomerular diseases [1–3]. The prevalence of ESRD in children in the United States is 8.3 per 100,000 [4] and 1.5/100,000 in Israel [5]. Kidney transplantation provides the best long-term results and optimum quality of life [6–10]. Because of the shortage of kidney sources in Israel, more than 75% of children waiting for a renal transplant are on dialysis for more than 2 years due to the shortage of available kidneys [5] placing them at high risk of dialysis-associated complication. Data on the long-term outcome of this patient group are scarce.

In children, peritoneal dialysis (PD) is preferred over hemodialysis (HD) in terms of quality of life, growth, and preservation of residual renal function [7]. Hemodialysis poses greater risks of access failure, vascular thrombosis, and obliteration of the great veins, which can be compromised for life [2, 6, 11]. In addition, multisystem involvement in ESRD in this population can lead to growth retardation [12, 13], cardiovascular complications [14, 15], and hematological complications [16–18].

The life span of children with ESRD is significantly lower than that of the age- and gender-matched general population. In studies of children on dialysis from Australia and New Zealand, survival rates were 85.7% at 3 years, 79% at 10 years, and 66% at 20 years [19]. Lower survival rates were found in children less than 12 months; 3-year survival for this age group was 68% in the North American Pediatric Renal Transplant Cooperative Study (NAPRTCS) annual report [20]. Similar results were reported in other studies from Netherlands [21, 22], with little change in more recent studies [3] despite significant progress made in renal replacement therapy. The most important risk factors for poor treatment outcome were younger age at onset of dialysis and type of nonrenal comorbidities [1, 3, 19, 20]. The leading causes of death in children on dialysis are cardiovascular disease and infections [6, 19, 21].

The aim of the present study was to evaluate the long-term outcome of a large cohort of pediatric patients on chronic dialysis in a single tertiary medical center. Attention was focused on mortality rate and causes of death.

## 2. Materials and Methods

The study cohort included all patients with ESRD maintained on dialysis for at least 3 months in the Dialysis Unit of Schneider Children's Medical Center of Israel from January 1995 through December 2013. The following data were retrospectively recorded from the medical files of each patient:* clinical parameters* such as cause of ESRD, age at diagnosis, associated diseases, age at initiation of dialysis, type of dialysis (PD, HD, and both), duration of dialysis, dialysis complications;* laboratory parameters* such as complete blood cell count and blood chemistry (iron status was evaluated by recommended parameters);* parameters related to treatment of chronic kidney disease* such as weight and height standard deviation scores (SDS) at dialysis onset and study end, use of recombinant growth hormone (rGH) therapy, blood pressure percentiles and SDS by gender, age and height percentile, presence of left ventricular hypertrophy (LVH) measured by echocardiography (mass index > 51 gr/m^2.7^), use and number of antihypertensive drugs, administration of recombinant erythropoietin (rEPO), and need for blood transfusions throughout treatment.

### 2.1. Definitions

#### 2.1.1. Exit Site/Tunnel Infection

 Exit site/tunnel infection was diagnosed by the presence of purulent drainage with negative peritoneal fluid culture [23].* Peritonitis* was diagnosed by the presence of at least 2 of the following criteria: cloudy effluent and/or abdominal pain or fever; effluent leukocyte count of >100 cells/mm^3^ with >50% neutrophils; bacterial growth in the peritoneal culture [24].

#### 2.1.2. Anemia

Anemia was diagnosed when hemoglobin level was less than 11 g/dL [18] or when there was a need for blood transfusions.

#### 2.1.3. Hypertension

Hypertension was diagnosed by either blood pressure values over the 95th percentile for age and heights at 3 visits or a need for antihypertensive therapy. Data were presented by SDS scores.

### 2.2. Outcome Measures

#### 2.2.1. Primary Outcome

Primary outcome was as follows: death or survival at the end of the study period, survival with successful kidney transplant, or continuing dialysis at the end of the study period.

#### 2.2.2. Secondary Outcome

Secondary outcome was as follows: dialysis complications (number of infections, number of access failures, and need for exchange), changes in growth, recorded in *z* scores, cardiovascular disease defined as *z* scores of hypertension, and LVH and anemia (percent of the population and hemoglobin level.)

Outcome was analyzed overall and by dialysis modality (PD versus HD), age at onset of dialysis (>5 years versus <5 years), and year of initiation of dialysis (1995–2003 versus 2004–2013).

### 2.3. Statistical Analysis

Data were analyzed using BMDP statistical software [25]. Analysis of variance (ANOVA) was used to compare continuous variables (biological parameters) between groups, with Bonferroni's corrections for multiple comparisons and Pearson's chi-square test for discrete variables (such as complications). Mann-Whitney nonparametric *U* test was used to analyze parameters such as number of blood pressure drugs. Comparing changes over time, we used ANOVA with repeated measures or Wilcoxon test, as appropriate. Survival curves were formulated according to the Kaplan-Meier method. Patient survival was calculated per 100 patient dialysis years. A *p* value of ≤0.05 was considered significant.

The study was approved by the Ethics Committee of Rabin Medical Center.

## 3. Results

### 3.1. Background Characteristics

One hundred and ten children met the study criteria, 63 boys (57.3%) and 47 girls (42.7%) of mean age 10.67 ± 5.27 years (range: 1 month–24 yrs.) at onset of dialysis. Twenty-three patients were <5 years and 87 > 5 years; Seventy-four patients (67%) were adolescents (age: over 12 years). Eighty-two patients (74%) were of Jewish and 28 (26%) were of Arab origin. Maintenance dialysis duration was as follows: median 1.46 years (range: 0.25–17.54 years) and mean 2.48 ± 3.02 years. Mean duration of follow-up was 13.5 years ± 5.84. The major causes of ESRD are shown in Table 1.

Table 2 shows clinical parameters of the patients analyzed according to dialysis modality. Significance was found only in patient mean age (*p* < 0.001). The later period (2004–2013) was characterized by a twofold increase in the proportion of patients treated with PD (15% versus 31% of all dialyzed patients).

### 3.2. Dialysis Access

*For PD*, a two-cuffed peritoneal catheter was inserted by a specialized pediatric surgeon in the operating room. Prophylaxis with Cefamezine was administered in all patients during the later period of the study (2004–2013). All the caregivers were instructed for appropriate performance of the procedure emphasizing aseptic techniques and were managed by automated PD.* For HD*, an arteriovenous graft or fistula was used in 44 patients (all of whom weighed > 20 kg, 52%) [23] and a central venous perm-cuffed catheter (Permacath [26]) in 41 (48%). Noncuffed central catheters were only used when there was an acute indication for dialysis and used as a bridge until a permanent access could be secured. Overall, 5 vascular access exchanges per 1000 patient-years were needed; in 35 patients (59% of patients on HD exclusively) there was no need for a change of access throughout the treatment course. The main reason for catheter removal was infection.

### 3.3. Dialysis Complications

#### 3.3.1. Exit Site

Exit site infection rate was 5.9 episodes per 1000 patient-years.

#### 3.3.2. Hemodialysis

At least one episode of exit site infection occurred in 22 patients on HD both exclusively and sequentially (25%). Bacteremia was documented at least once in 19 patients on HD (22%). The average bacteremia rate was 4.4 episodes per 1000 patient-years.

#### 3.3.3. Peritoneal Dialysis

16 patients developed exit site infection (31%). The average peritonitis rate was 20 episodes per 1000 patient-years. Tunnel infections were 2.2 episodes per 1000 patient-years. No bacteremia episodes were noted in patients on PD.

The rate of total catheter-related infections was significantly higher for PD than for HD (*p* < 0.001). The earlier treatment period was characterized by a significantly higher rate of exit site infections (68.8% versus 28%, *p* < 0.001) and of bacteremia (19.1% versus 40.8%, *p* < 0.02). The peritonitis rate in the patients on PD was also higher in the earlier period, but the difference did not reach statistical significance (68% versus 81%, *p* = 0.35).

### 3.4. Anemia

Anemia was documented in 101 patients (92%) before onset of dialysis. Mean hemoglobin level was 9.11 ± 1.69 gr/dL at onset of dialysis and 10.66 ± 1.52 gr/dL (*p* < 0.001) at study end. The rate of rEPO administration was 85.3% at onset of dialysis, increasing to 97.8% at study end (*p* = 0.01).

Findings by dialysis modality are shown in Table 2. Blood transfusion was necessary in 21 patients (19.8%) during the first 3 months on dialysis and in 5 patients (4.5%) during the last 3 months on dialysis.

### 3.5. Growth and Nutrition

Mean weight SDS was −0.56 ± 0.84 at dialysis onset and −0.84 ± 0.69 at study end (*p* = 0.01). Respective values for height were −1.22 ± 1.11 and −1.62 ± 1.26; this difference was not statistically significant (*p* = 0.13). Growth parameters analyzed by dialysis modality are shown in Table 2. Patients were placed on an age- and weight-adjusted diet under careful follow-up by a renal dietitian. A gastrostomy tube was inserted in 13 cases (14%). Forty-seven patients (43%) were treated with rGH.

### 3.6. Hypertension

Seventy-seven patients (70%) were hypertensive (systolic, diastolic, or both) at onset of dialysis. Mean systolic blood pressure SDS decreased from 3.35 ± 2.19 at onset of dialysis to 2.14 ± 2.83 at study end (*p* < 0.001). Respective values for diastolic blood pressure SDS were 2.51 ± 1.72 and 1.36 ± 2.22 (*p* < 0.001). The number of medications needed to control hypertension ranged from 1 to 4 (median 1) at onset of dialysis and decreased to 0 to 5 (median 0) at study end. Seventy patients (63%) needed treatment at dialysis onset compared to 46 (42%) at study end (*p* < 0.001).

LVH was found in 50 patients (46%) at onset of dialysis and 37 (34%) at study end (*p* = 0.01). During treatment, LVH developed in 11 patients (10.5%) who had not had LVH at dialysis onset.

### 3.7. Outcome

Eighty-nine patients (90.3%) received a kidney graft during the study period, of whom 79 (88.8%) had a functioning graft at study end and 10 (11.2%) lost graft function and returned to dialysis. Causes for loss of graft included 5 patients with noncompliance; 1 with FSGS recurrence; 2 with acute rejection; 1 with graft renal artery thrombosis; 1 with chronic antibody mediated rejection. Eighty-six patients had one renal transplant and 2 patients had 2 transplants. Sixty-four grafts originated from deceased donors, 15 were from living-related donors, and 9 were from living nonrelated donors. Another 8 patients (7.3%) continued dialysis; this group age was on average 11 ± 6.4 years at onset of dialysis and were on maintenance dialysis for an average of 2.85 ± 3.9 years (range: 0.25–6.64). One child with CAKUT ceased dialysis with stable kidney function.

12 patients died during the study period, with a mortality rate of 10.9%. Causes of death are detailed in Table 3. Two patients who died suffered from Schimke immunoosseous dysplasia complicated by moyamoya phenomenon. Their death was attributed to cerebrovascular accidents. Three patients who died from hyperkalemia were on Kayexalate and a low potassium diet.

**Figure 1:**
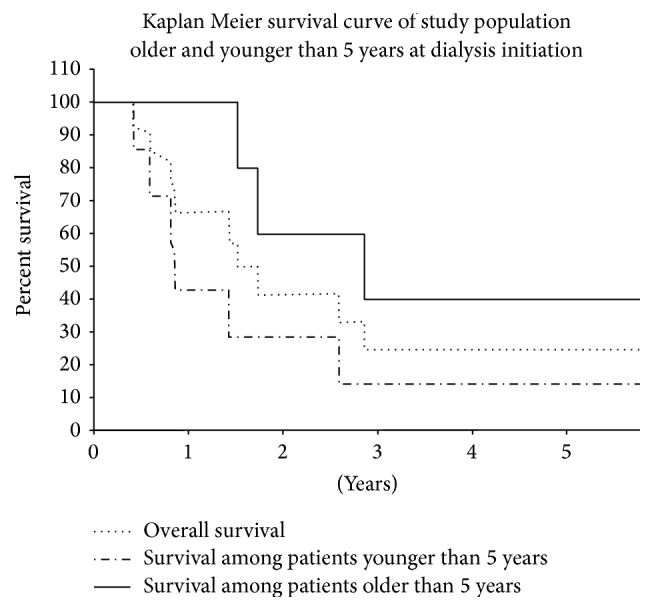
Kaplan Meier survival curve of study population and Kaplan Meier survival curves of children beginning dialysis at age > 5 years or <5 years.

On Kaplan-Meier analysis, the 5-year overall survival rate on dialysis was 84%: 90% in children > 5 years and 61% in children < 5 years at dialysis onset (Figure 1). Patients treated with PD exclusively had survival rates of 100% at 1 year and 78% at 5 years; the rate for HD exclusively was 93% at both time points and for combined modalities 95% and 68%. There was no statistically significant difference in overall survival among the groups (*p* = 0.56).

Mortality rates analyzed by patient-years on dialysis were 4.3 deaths per 100 patient-years for the whole cohort, 2.39 deaths per 100 patient-years for patients > 5 years at onset of dialysis, and 11.6 deaths per 100 patient-years for patients < 5 years at onset of dialysis. The mortality rate for PD exclusively was 9 per 100 patient-years, for HD exclusively was 7.26 per 100 patient-years, and for combined treatment was 3.93 per 100 patient-years. By treatment period, 5-year survival rates were 73% in patients treated in 1995–2003 and 90% in patients treated in 2004–2013; the difference was not statistically significant (*p* = 0.81).

## 4. Discussion

To the best of our knowledge, this is one of the largest single-center longitudinal outcome series of paediatric ESRD managed with long-term maintenance dialysis in the medical literature. Previous studies described smaller cohorts of 34 [27] and 98 children [8] with shorter follow-up times of 9 and 14 years, respectively. Males were slightly overrepresented in our study (57.3% versus 42.7% for females), in agreement with findings that ESRD is more common in males [6, 21, 22, 24, 25]. Also expected was the younger age of patients who started with PD compared to patients who started with HD (7.58 versus 12.41 years) given the considerable technical difficulties with dialysis in younger children [2, 11].

Maintenance dialysis duration in our study, median 1.46 years, range 0.25–17.54 years, and mean 2.48 ± 3.02 years, was similar to that in centers in Europe and USA [6, 8, 28] despite the limited graft sources in a small country such as Israel. The limited amount of potential donors in Israel can be explained by various religious beliefs causing individuals not to donate organs.

Our dialysis unit can provide both HD and PD. PD is known to have several advantages over HD in children, including better preservation of native renal function, lack of long-term compromise of the main venous vascular tree, and freedom from frequent hospital visits with significantly less interference with everyday life activities and quality of life. Nevertheless, only 35% of our patients were started on PD compared to 80% in other series [8]; this can be explained by the reasonable travel distances to the pediatric dialysis unit from most parts of our country; therefore patients who cannot be managed with PD at home are easily switched to HD when it is in their best interest. Furthermore, 67% of our cohort were adolescents, many of whom may have declined PD because of its major impact on body image.

Primary graft/arteriovenous fistulas are considered the best permanent vascular access for HD with lowest risks of secondary failure and complications [26]; they were used in 52% of our patients, a significantly higher rate than reported in the literature (36% and 21.3%) [22, 28]. The rate of infections was low in our study compared to NAPRTCS [20]: 2 episodes per 100 patient-years versus 8.1 per 100 patient-years. Low rates of secondary failure and infection rates have been associated with high surgical skills and expertise and use of standardized PD education programs, administration of prophylactic antibiotics prior to insertion of PD catheters, and aseptic techniques.

Anemia may be caused by a multitude of factors and poses a challenging problem in patients on chronic dialysis similar to other studies. Ninety-two percent of our patients were anemic at onset of dialysis, with a mean hemoglobin level of 9.11 ± 1.69 gr%. By the end of the study, this value increased significantly to 10.66 ± 1.52 gr% (*p* < 0.001), concomitant with a decrease in the need for blood transfusions (from 19.8% of patients to 4.5%; *p* < 0.001). A similar positive trend was found in the annual NAPRTCS report with a hematocrit range of 29.9% to 32% at onset of dialysis, which improved after 6 months to 32.3%–33% as found in [20]. It is possible that the more intensive rEPO treatment given during dialysis compared to the predialysis period was responsible for this finding. In addition, we suspect that compliance was better for intravenous than subcutaneous rEPO administration.

Cardiovascular disease is a major cause of death in adolescents and young adults [15]. In their nationwide Dutch study partly focused on cardiovascular disease, Groothoff et al. [15] reported LVH rates of 47% and 39% in male and female adolescents, respectively, and in an analysis of a large European registry, Fadrowski et al. [16] found that uncontrolled hypertension was present in 45.5% of patients on HD and 35.5% on PD; rates of antihypertensive drug use in these groups were 69.7% and 68.2% male/female, respectively, and in 51.9% of all patients. In our study, 70% of children presented with hypertension at onset of dialysis. By study end, there was a significant improvement in blood pressure SDS, with a decrease in the LVH rate from 46% to 34% (*p* = 0.01). Improvement in LVH over time on dialysis can be explained by good fluid volume control and close monitoring of cardiovascular parameters and better patient compliance and adherence to treatment.

Growth retardation is a complication of chronic kidney disease and has a significant influence on both final adult height and quality of life [7, 13]. In the NAPRTCS reports, growth rate improved significantly, from −2.8 to −1.9 (*p* = 0.078), in patients treated with rGH (9.4% of patients on PD and 8.7% of patients on HD) but worsened in the remainder [8, 20]. We did not observe a significant improvement in growth in the present study, perhaps our patients were not as growth retarded initially as in the NAPRTCS data which may explain their relative lack of response to GH. Also some of our patients may have already reached their adult height.

According to the NAPRTCS reports [20, 22] survival in paediatric patients on dialysis, calculated by deaths per 100 patient-years, varied from 13.6 at age 1 year, to 8.2 at age 1-2 years, 6.1 at age 2–5 years, and 2.8 at age > 6 years. Overall survival rates were 95% at 1 year and 85.7% at 3 years [20, 22]. In the long-term study from Australia and New Zealand by McDonald and Craig [19], 10-year and 20-year survival rates of children on dialysis were 79% and 66%, respectively. The United States Renal Data System (USRDS) study by Mitsnefes et al. [3] reported an overall mortality of 9.88 per 100 patient-years in patients younger than 5 years and 3.86 per 100 patient-years in patients older than 5 years. Shroff et al. [8] in a single-Center 14-year study reported 17 deaths in 98 patients, for an overall survival rate of 83%. In our present 18-year study, of 110 young patients on maintenance dialysis, 12 died. Eight deaths (7.2%) were related to dialysis complications. The 5-year overall survival rate was 84%: 61% in patients younger than 5 years and 90% in patients older than 5 years at onset of dialysis. Calculating survival in patient-years of dialysis yielded a better outcome than in the study of Mitsnefes et al. [3]: 4.3 deaths for the whole cohort, 11.6 in the younger group, and 2.39 in the older group. Only 2 patients who died were exclusively on PD and those 2 died due to complications of their primary illness. It was not found statistically significant, probably because of a small number of deaths.

The limitation of the present study is its retrospective design, a small sample size which for a single center is relatively large but not compared with multicenter studies, and a specific population which may defer from other centers/groups.

In conclusion, despite the relatively long period on maintenance dialysis, pediatric patients with ESRD in our center have a similar outcome in terms of survival and dialysis complications to that reported in other industrialized countries. We suggest that efforts be made to broaden the use of arteriovenous fistulas and minimize the use of central cuffed catheters in order to avoid infections and thrombosis. Implementation of standardized PD education programs, prophylactic antibiotics prior to insertion of PD catheters, and strict adherence to aseptic techniques may reduce the rate of PD -associated infections.

The findings may have important implications for decreasing the risks of dialysis-associated complications especially for very young ESRD patients, thereby lowering hospitalization and improving survival.

## Figures and Tables

**Table 1:** Causes of ESRD leading to need for dialysis.

Major causes of ESRD	Number (%)
CAKUT	45 (40.9%)
Nephronophthisis	6 (5.45%)
FSGS	19 (17.27%)
Congenital nephrotic syndrome	4 (3.63%)
Metabolic diseases	5 (4.54%)
Alport	2 (1.81%)
Denys-Drash syndrome	3 (2.72%)
Glomerulonephritis	12 (10.9%)
Hypoxic injury	1 (0.9%)
Familial HUS	2 (1.81%)
Secondary HUS	2 (1.81%)
PCKD	2 (1.81%)
Other/unknown	7 (6.36%)

**Table 2:** Study parameters related to dialysis modality.

Parameters	HD exclusively (*n* = 59, 53.6%)	PD exclusively (*n* = 25, 22.7%)	Both modalities ^*∗*^ (*n* = 26, 23.6%)	*p* value
Age at dialysis onset (yr.), mean ± SD	12.41 ± 4.58^†^	7.58 ± 5.36^†^	9.68 ± 5.21	<0.001
Duration of dialysis (yr.), mean ± SD	1.41 ± 0.79	1.32 ± 0.47	1.41 ± 0.76	0.87
Number of patients by period of treatment, *n* (%)				
1995–2003	33 (55.9%)	9 (15.3%)	17 (28.8%)	0.09^‡^
2004–2013	26 (51.0%)	16 (31.4%)	9 (17.6%)	
Hb level, g/dL, mean ± SD				
At dialysis onset	9.18 ± 1.62	9.22 ± 1.61	8.8 ± 1.96	0.6
At study end	10.88 ± 1.41	10.55 ± 1.75	10.32 ± 1.46	0.3
Growth SDS, mean ± SD				
At dialysis onset				
Height SDS	−1.25 ± 0.94	−1.01 ± 1.50	−1.4 ± 0.83	0.52
Weight SDS	−0.64 ± 0.70	−0.31 ± 1.20	−0.55 ± 0.64	0.25
At study end				
Height SDS	−1.47 ± 1.22	−1.46 ± 1.44	−2.3 ± 0.8^†^	0.009
Weight SDS	−0.81 ± 0.62	−0.37 ± 0.79	−1.22 ± 0.46	<0.001

^*∗*^PD and HD sequentially. ^†^ *p* < 0.001. ^‡^The number of patients treated by PD increased significantly in the later period.

**Table 3:** Characteristics of patients on maintenance dialysis who died during the study period (12/110).

Pt. number	Age at dialysis initiation (years)	Sex	Cause of ESRD	Duration of dialysis (years)	Modality of dialysis	Access	Age at death (years)	Cause of death
1	1.58	M	Familial HUS	0.86	HD	P	2.42	Cardiac arrest due to hyperkalemia
2	3.69	M	CAKUT	2.35	Combined	P + T	6.04	Sepsis
3	1.98	M	HUS s/p BMT	0.42	Combined	P + T	2.41	Sepsis
4	2	F	Bilateral Nephrectomy due to Wilms tumor	0.59	Combined	P + T	2.59	Cardiac arrest due to hyperkalemia
5	3.54	F	Familial HUS	9.57	HD	AVF	13.11	Access failure
6	14.51	F	Systemic Lupus Erythematosus	6.23	HD	AVF	20.74	Mesenteric event
7	7.96	M	FSGS	1.52	Combined	P + T	9.48	Access failure
8	7.8	M	FSGS-Schimke syndrome	2.86	PD	T	10.66	CVA
9	9.08	M	FSGS-Schimke syndrome	1.73	PD	T	10.81	CVA
10	3.2	M	Congenital nephrotic syndrome	0.81	HD	P	4.01	Cardiac arrest due to hyperkalemia
11	9.55	F	Nephrotoxic kidney injury	5.91	HD	AV	15.46	Metastatic Neuroblastoma
12	4.62	M	CAKUT	1.43	Combined	P + T	6.05	Sepsis

AVF: arterial venous fistula; BMT: bone marrow transplantation; CAKUT: congenital anomalies of the kidney and urinary tract; CVA: cerebrovascular accident due to moyamoya phenomenon; ESRD: end-stage renal disease, FSGS: focal segmental glomerulosclerosis; HD: hemodialysis, HUS: hemolytic uremic syndrome; P: permacath; PD: peritoneal dialysis; s/p: status post; T: tenckhoff peritoneal catheter.
